# Multidimensional Roles of Deubiquitinases in Tumor Glycolytic Reprogramming

**DOI:** 10.3390/ijms27219856

**Published:** 2026-11-03

**Authors:** Jiawei Zhao, Zhicai Zhao, Shiqi Zang, Xiaotong Guo, Jingyi Zhang, Jinfeng Yang, Xingyu Mu, Bin Li, Jingjing Du, Gang Xu, Lin Qi, Juzheng Zhang, Jian Chen, Jiamin Jin, Zuyang Ye

**Affiliations:** 1 Department of Nephrology, The Second Affiliated Hospital of Guilin Medical University, Guilin 541199, China; 13256566591@163.com; 2 Key Laboratory of Tumor Immunology and Microenvironmental Regulation, Guilin Medical University, Guilin 541199, China; 15207860664@163.com (Z.Z.); keepgoingwendy@163.com (S.Z.); guoxiaotong@stu.glmu.edu.cn (X.G.); zhang_jingyi2002@163.com (J.Z.); gmuyangjf@glmc.edu.cn (J.Y.); mxy19920322@glmu.edu.cn (X.M.); 212020524@glmu.edu.cn (G.X.); qilin@glmu.edu.cn (L.Q.); chenjian@glmc.edu.cn (J.C.); 3 School of Basic Medical Sciences, Guilin Medical University, Guilin 541199, China; l1498475943@163.com (B.L.); 15588908521@163.com (J.D.)

**Keywords:** deubiquitinases, glycolysis, metabolic reprogramming, signaling pathways, tumor microenvironment, targeted therapy

## Abstract

Tumor glycolytic reprogramming is an important metabolic hallmark of malignant tumors and plays a key role in sustaining tumor cell proliferation, adapting to microenvironmental changes, and promoting tumor progression. In recent years, deubiquitinating enzymes (DUBs), as important regulators of post-translational protein modification, have attracted extensive attention for their roles in tumor glycolytic reprogramming. Studies have shown that DUBs can not only directly affect glycolysis by regulating the stability and functional status of key glycolytic enzymes, glucose transporters, and upstream transcriptional regulators, but also indirectly regulate glucose metabolism through signaling pathways such as AMPK, mTOR, HIF-1, and PI3K/AKT. In addition, DUBs can participate in the interplay between tumor metabolism and the immune microenvironment by affecting lactate production in tumor cells, metabolic adaptation of immune cells, and immune checkpoint signaling. Notably, glucose metabolism-related products and changes in metabolic status can also reciprocally regulate the expression and function of DUBs through mechanisms such as lactylation, O-GlcNAcylation, and phosphorylation, suggesting that bidirectional feedback regulation may exist between the two. Drugs targeting DUBs have shown potential to interfere with glycolysis and inhibit tumor progression in some preclinical tumor models; however, their clinical translation still faces challenges, including context-dependent mechanisms of action, inhibitor selectivity, and safety. This article systematically reviews the molecular mechanisms by which DUBs regulate tumor glycolytic reprogramming, with a focus on their roles in metabolic signal transduction, interactions with the tumor immune microenvironment, and metabolic feedback regulation, and summarizes the research progress and existing issues in related targeted intervention strategies, aiming to provide new insights for mechanistic studies of tumor metabolic reprogramming and precision therapy.

## 1. Introduction

Glucose metabolism is a core biological process that maintains cellular energy supply, biosynthesis, and metabolic homeostasis, and it plays important roles in cell growth, proliferation, and stress adaptation. Glucose is not only an important energy substrate for the generation of adenosine triphosphate (ATP), but also provides carbon sources and metabolic intermediates for the biosynthesis of nucleic acids, lipids, amino acids, and glycoconjugates through different metabolic pathways [1]. In addition, glucose metabolism participates in cellular redox homeostasis, post-translational protein modifications, and signal transduction. In mammalian cells, after glucose enters cells via glucose transporters, it can generate pyruvate through glycolysis; pyruvate can be further converted to lactate or enter mitochondria and participate in energy metabolism through the tricarboxylic acid cycle (TCA cycle) and oxidative phosphorylation (OXPHOS) [2,3]. Meanwhile, glucose and glycolytic intermediates can also be shunted into branches such as the pentose phosphate pathway (PPP), hexosamine biosynthetic pathway (HBP), serine synthesis pathway (SSP), and glycogen metabolism, providing material basis for nucleotide synthesis, redox balance, protein glycosylation, and other anabolic processes [2,4,5]. These metabolic pathways are closely connected through shared metabolic intermediates and multi-level regulatory networks, enabling cells to dynamically adjust glucose utilization according to energy demands, nutrient status, and environmental changes.

Tumor metabolic reprogramming is an important biological hallmark of malignant tumors; by altering nutrient uptake, metabolic pathway activity, and metabolic resource allocation, it meets the demands of sustained proliferation, biosynthesis, and microenvironmental adaptation of tumor cells [6]. Among these, glucose metabolic reprogramming is one of the most representative metabolic alterations, typically manifested as increased glucose uptake, remodeled glycolytic flux, enhanced lactate production, and redistribution of glucose-derived carbon flux [3]. The Warburg effect is a classic manifestation of this process, whereby many tumor cells maintain high levels of glycolysis and lactate production even under sufficient oxygen conditions. This metabolic mode can support the rapid proliferation of tumor cells by providing carbon sources required for nucleotide, lipid, and amino acid synthesis [1]. Although the ATP yield per molecule of glucose from glycolysis is relatively low, high glycolytic flux still helps to rapidly meet energy demands [7]. Acidification of the microenvironment caused by lactate efflux can promote extracellular matrix degradation, immune suppression, and invasion and metastasis, while regulation of redox homeostasis helps enhance tolerance to oxidative stress [8].

Tumor glycolytic reprogramming is regulated at multiple levels by metabolic enzymes, oncogenic signaling pathways, and transcriptional regulatory networks. At the metabolic execution level, key proteins such as glucose transporter 1 (GLUT1), hexokinase 2 (HK2), PFKFB3, pyruvate kinase M2 (PKM2), and lactate dehydrogenase A (LDHA) jointly participate in glucose uptake, regulation of glycolytic flux, and lactate production [3]. At the signal transduction level, pathways such as PI3K/AKT/mTOR and RAS/MAPK regulate the expression and metabolic activity of glycolysis-related proteins by coordinating cell proliferation signals with metabolic demands [5,9,10]. At the transcriptional regulation level, hypoxia-inducible factor-1α (HIF-1α) can induce the expression of glycolysis-related genes and, by upregulating pyruvate dehydrogenase kinase 1 (PDK1), restrict pyruvate from entering mitochondrial oxidative metabolism; transcriptional regulators such as MYC and p53 affect glucose utilization, anabolism, and redox homeostasis through different mechanisms [11,12,13]. These regulatory mechanisms intersect with one another and jointly shape a dynamic and heterogeneous tumor glycolytic phenotype.

In addition to the expression of metabolic enzymes and regulation by classical signaling pathways, post-translational modifications (PTMs) are also important molecular mechanisms affecting tumor glycolytic reprogramming. Among them, ubiquitination, as a dynamic and reversible protein modification, not only participates in protein degradation but also regulates protein activity, subcellular localization, and intermolecular interactions through non-degradative ubiquitin signals, and can exert differential functions through different modification forms: monoubiquitination is generally not a typical proteasomal degradation signal and mainly regulates protein activity and signal transduction by modulating substrate protein conformation, subcellular localization, and intermolecular interactions; polyubiquitination produces different biological effects depending on the ubiquitin chain linkage sites, with K48-linked polyubiquitin chains mainly mediating proteasome-dependent degradation of substrates, whereas K63-linked polyubiquitin chains mostly exert non-degradative regulatory roles and participate in processes such as protein kinase activation and complex assembly [14,15,16,17]. Deubiquitinating enzymes (DUBs) finely regulate protein stability and functional status by removing or trimming ubiquitin modifications on substrate proteins, thereby participating in various cellular biological processes [18]. In recent years, increasing studies have shown that DUBs can not only directly regulate the stability or catalytic activity of glycolysis-related enzymes, but also participate in tumor glycolytic reprogramming at different regulatory levels by affecting the functions of HIF-1α, MYC, and related signaling molecules. For example, USP25 can enhance glycolytic activity and hypoxia adaptation in pancreatic cancer cells by deubiquitinating and stabilizing HIF-1α [19]. In addition, glycolytic reprogramming is not confined to tumor cells; the resulting glucose competition, lactate accumulation, and local metabolic environment changes can also affect the metabolic status and function of immune cells in the tumor microenvironment, thereby participating in shaping the tumor immune microenvironment. It is noteworthy that metabolites produced by glycolysis are not merely end products or intermediates of metabolic reactions; they can also reversely affect metabolic regulatory networks through mechanisms such as signal transduction and post-translational modification of proteins, forming dynamic feedback. Previous studies have found that PSMD14 can promote glycolysis and lactate accumulation by regulating PFKFB2 and, through histone lactylation, form a positive feedback loop that promotes its own expression [20]. These findings suggest that DUB regulation of tumor glycolysis may extend beyond a single metabolic enzyme or signaling pathway and involve complex interactions between intracellular metabolic regulation and metabolite feedback.

Although previous studies have elucidated the roles of DUBs in tumor glycolytic reprogramming from the perspectives of metabolic enzymes, signaling pathways, and transcriptional regulators, current understanding is still mainly centered on specific molecules or regulatory pathways. The functional links between different regulatory mechanisms and specific glucose metabolic processes, as well as the interactions between these metabolic changes and the tumor immune microenvironment, remain to be further integrated and clarified [21,22,23]. In addition, the potential reverse regulation of DUB activity and function by cellular metabolic status, as well as the context-dependent roles of different DUBs in specific tumor types and microenvironmental conditions, also warrant in-depth investigation. Therefore, building on previous studies, this review systematically integrates the molecular mechanisms and relevant research evidence of DUBs involved in tumor glycolytic reprogramming from four interrelated dimensions: regulation of glycolysis-related proteins, metabolic signaling pathways, interactions with the tumor immune microenvironment, and metabolic feedback. This review focuses on the links between different regulatory mechanisms and specific metabolic processes, distinguishes experimentally validated metabolic and immune regulatory effects from potential regulatory relationships that remain to be confirmed, and further discusses the reverse effects of metabolic status on DUB function and the possible feedback regulation formed thereby. On this basis, we further analyze the potential value, current challenges, and future research directions of DUBs as targets for tumor metabolic intervention, aiming to provide a more comprehensive and critical perspective for understanding DUB-mediated tumor glycolytic reprogramming.

## 2. Regulation of Tumor Glycolysis-Related Proteins by DUBs

**Figure 1:**
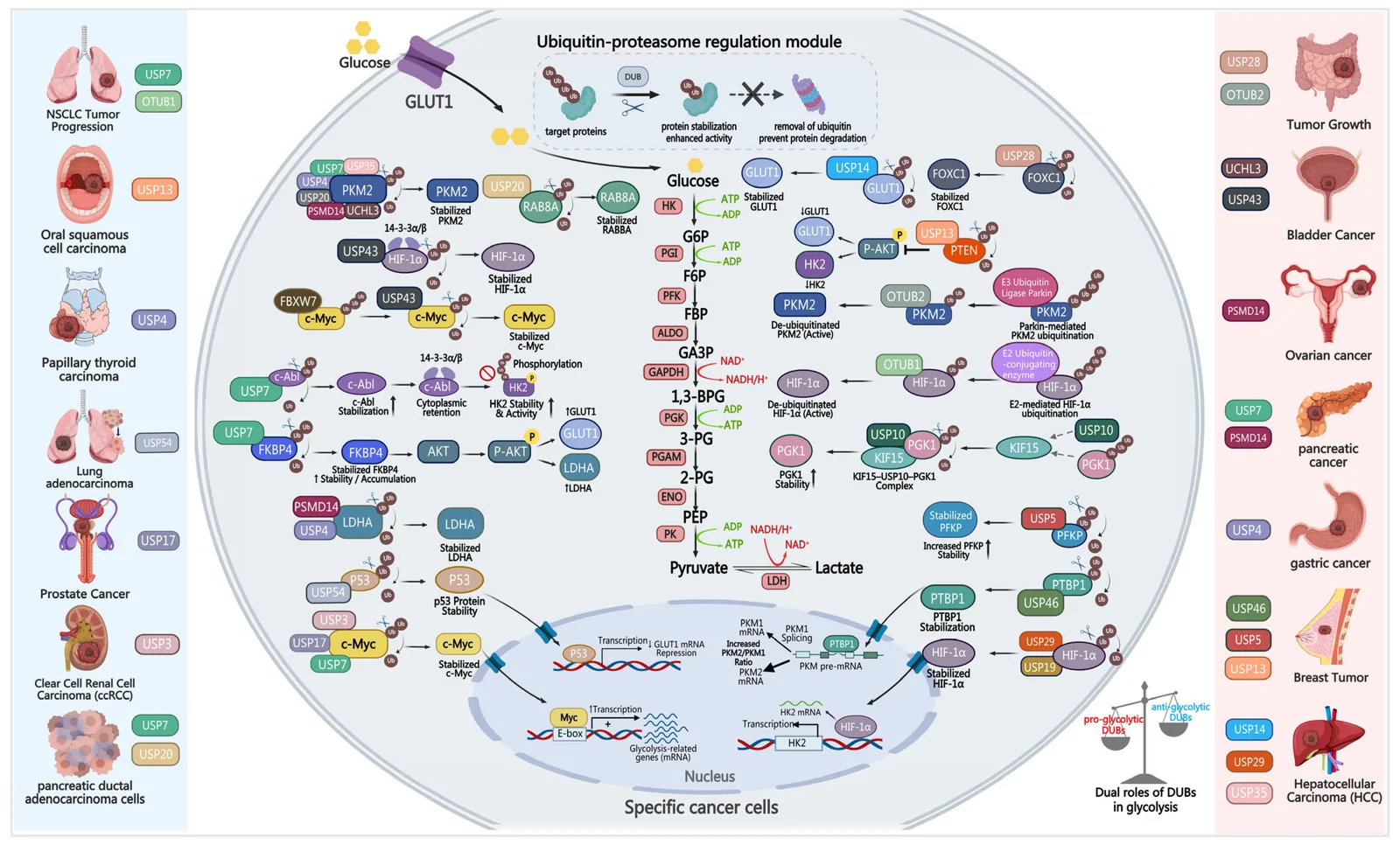
Molecular mechanisms underlying the regulation of glycolysis-associated proteins by deubiquitinases in cancer. DUBs regulate tumor glycolytic reprogramming through multiple mechanisms, including the modulation of glycolytic enzyme stability and activity, glucose transporter trafficking, and the functions of metabolic transcription factors and signaling regulators. These effects involve the removal of degradative or non-degradative ubiquitin chains, as well as non-catalytic mechanisms that influence protein interactions and substrate stability. Depending on their substrates, regulatory mechanisms, and tumor contexts, DUBs may either promote or suppress glycolysis by altering glucose uptake, glycolytic enzyme activity, and metabolic gene expression. The schematic summarizes representative DUB-mediated regulatory pathways reported in different cancer models. In the glycolytic pathway, green arrows indicate ATP/ADP interconversion, while red arrows indicate NAD^+^/NADH interconversion. Created with MedPeer (medpeer.cn). Certificate No.: 71570fq2sghzw5ojv1793423049.

DUBs play important roles in tumor glycolytic reprogramming by regulating the ubiquitination status, stability, and biological activity of glycolysis-related proteins. As illustrated in Figure 1, glucose enters tumor cells through glucose transporters and undergoes glycolysis to generate pyruvate, a process involving ATP consumption and production and NAD^+^/NADH interconversion. Pyruvate can subsequently be converted into lactate, thereby regenerating NAD^+^ to sustain glycolysis. Within this metabolic framework, DUBs exert regulatory effects at multiple levels, including direct deubiquitination of key glycolytic enzymes and glucose transporters, indirect modulation of metabolic transcription factors and signaling molecules, and non-canonical mechanisms independent of their catalytic activity. These regulatory mechanisms may influence glucose uptake, glycolytic enzyme activity, and lactate production, thereby contributing to tumor metabolic adaptation in a context-dependent manner (Figure 1).

### 2.1. Direct Deubiquitination of Glycolytic Enzymes

Glycolysis is an important component of tumor metabolic reprogramming, and its activity is determined not only by the expression levels of metabolic enzymes but also by protein stability, catalytic status, and subcellular localization. DUBs can reverse ubiquitination modifications on metabolic enzymes and thereby regulate key glycolytic nodes at the post-translational level. Current evidence indicates that DUBs regulate glycolytic enzymes mainly through the maintenance of protein stability, coordination of multi-enzyme complexes, regulation of non-degradative ubiquitination, and metabolite-mediated signaling.

Maintaining the protein stability of key metabolic enzymes is an important mechanism by which DUBs promote tumor glycolysis. In triple-negative breast cancer (TNBC), USP5 directly binds to and deubiquitinates PFKP, inhibits its protein degradation, and enhances PFKP stability, thereby maintaining glycolytic activity and promoting tumor progression. USP5 knockdown increases PFKP ubiquitination, accelerates its protein degradation, and inhibits TNBC cell proliferation and tumor growth in vivo, suggesting that the USP5–PFKP axis has potential therapeutic value [24]. PKM2 is a representative substrate regulated by multiple DUBs. The interaction between USP20 and PKM2 and the regulation of K48-linked ubiquitination of PKM2 by USP7 provide initial molecular evidence for its deubiquitination-mediated regulation [25,26]. Further studies have shown that USP4 in gastric cancer, USP35 in hepatocellular carcinoma, and UCHL3 in bladder cancer maintain PKM2 protein stability by inhibiting ubiquitin-dependent degradation and promote related malignant phenotypes [27,28,29]. In addition, in papillary thyroid carcinoma, USP4 maintains LDHA stability by inhibiting its proteasomal degradation, and this effect is associated with MAPK and AKT signaling activation [30]. These studies indicate that DUBs can maintain the protein abundance of metabolic enzymes at multiple glycolytic nodes; however, their contribution to actual glycolytic flux still requires functional metabolic analysis.

DUBs can also coordinate glycolysis-related metabolic processes by regulating multi-enzyme complexes or auxiliary protein recruitment. JOSD2 deubiquitinates and regulates metabolic enzymes such as ALDOA, PFK1, and PHGDH, maintaining their stability and activity and thereby promoting glucose metabolism and tumor cell proliferation. Because PHGDH participates in shunting glycolytic intermediates toward the serine biosynthetic pathway, JOSD2 may link glycolysis with biosynthetic demands by coordinating the protein homeostasis of different metabolic enzymes [31]. In pancreatic cancer, KIF15 acts as a molecular scaffold and recruits USP10 and PGK1 to promote PGK1 deubiquitination and protein stabilization, thereby enhancing glycolysis and cell proliferation [32]. These findings suggest that the regulation of metabolic enzymes by DUBs depends not only on catalytic activity but also on protein complex assembly and substrate recruitment efficiency.

In addition to reversing degradative ubiquitination, DUBs can alter the functional state of glycolytic enzymes by regulating non-degradative ubiquitin chains. In ovarian cancer, PSMD14 reduces K63-linked ubiquitination of PKM2 and, without significantly affecting its protein abundance, decreases PKM2 tetramer formation, reduces its pyruvate kinase activity, and promotes nuclear translocation, which is associated with enhanced aerobic glycolysis and malignant tumor progression [33]. This mechanism differs from those of USP4, USP7, USP35, and UCHL3, which mainly affect PKM2 stability by regulating degradative ubiquitination. Because the oligomeric state and nuclear localization of PKM2 are also involved in the allocation of metabolic intermediates and the regulation of gene expression, a decrease in its catalytic activity does not necessarily indicate attenuation of the overall glycolytic phenotype. This indicates that the effects of DUBs on metabolic enzymes depend not only on protein expression levels but also on the type of ubiquitin chain and the functional state of the substrate.

The direct regulation of glycolytic enzymes by DUBs can further affect epigenetics and other metabolic pathways through metabolites. In pancreatic cancer, PSMD14 stabilizes LDHA by deubiquitination, promotes lactate accumulation, and thereby enhances histone H3K18 lactylation (H3K18la), upregulates the transcriptional expression of ATP citrate lyase (ACLY), and promotes fatty acid synthesis, thus driving tumor progression. This PSMD14–LDHA–H3K18la–ACLY axis reveals a functional link between glycolysis, epigenetics, and lipid metabolism [34]. Notably, PSMD14 mainly regulates the non-degradative ubiquitination of PKM2 to affect its functional state in ovarian cancer, whereas it stabilizes LDHA to promote lactate-mediated downstream signaling in pancreatic cancer, suggesting that the same DUB may exert differential metabolic effects depending on substrate selection, ubiquitin chain type, and tumor context.

In summary, DUBs participate in tumor glycolytic reprogramming by maintaining the protein stability of glycolytic enzymes, coordinating multi-enzyme complex function, altering the catalytic state of metabolic enzymes, and regulating metabolite-mediated signaling, and may connect glycolysis with other biosynthetic pathways.

### 2.2. Regulation of Glucose Transport

Glucose uptake is a key step for tumor cells to maintain glycolytic activity and metabolic reprogramming, and is mainly mediated by glucose transporters (GLUTs), among which GLUT1 and GLUT3 are aberrantly expressed in various malignant tumors [35]. In addition to expression levels, the protein stability and membrane localization of GLUTs also affect glucose uptake capacity. Studies have shown that TXNIP can promote GLUT1 endocytosis and mediate its lysosomal trafficking through subsequent ubiquitination modifications [36]. Therefore, deubiquitinating enzymes (DUBs) can participate in the regulation of tumor glucose uptake by modulating GLUT protein turnover, membrane trafficking, and upstream signaling networks.

With respect to the direct regulation of transporter stability, studies in hepatocellular carcinoma have shown that USP14 removes K48-linked polyubiquitin chains from GLUT1, inhibits its proteasomal degradation, and enhances glucose uptake. Site-directed mutagenesis experiments indicated that K245 is an important site for K48-linked polyubiquitination of GLUT1. USP14 deficiency decreased GLUT1 protein levels and glucose uptake, whereas GLUT1 re-expression partially reversed its tumor growth inhibitory effect, indicating that USP14 promotes tumor metabolic adaptation by directly stabilizing GLUT1 [37]. In contrast, USP20 indirectly affects GLUT1 membrane localization by regulating vesicular trafficking. Studies in pancreatic ductal adenocarcinoma have found that USP20 selectively removes K48-linked polyubiquitin chains from GDP-bound inactive RAB8A, promoting its accumulation and interfering with the normal GTP/GDP cycle, thereby limiting GLUT1 trafficking to the plasma membrane, reducing glucose uptake and lactate production, and inhibiting tumor cell proliferation and metastasis [38]. These two opposite metabolic effects indicate that the regulatory roles of DUBs depend on the molecular function and activation state of the substrate. In addition, some DUBs indirectly regulate glucose uptake through PI3K/AKT-related signaling networks. Studies in breast cancer have demonstrated that USP13 can directly deubiquitinate and stabilize PTEN, inhibiting AKT activation and glycolysis; further studies in oral squamous cell carcinoma have found that USP13 downregulates GLUT1 and HK2 expression through the PTEN/AKT signaling pathway, reducing glucose uptake and lactate production [39,40]. Similarly, in non-small cell lung cancer with acquired osimertinib resistance, USP7 stabilizes FKBP4 through deubiquitination, participates in PI3K/AKT-related metabolic regulation, and is associated with upregulated GLUT1 and LDHA expression and enhanced glycolysis [41]. However, current studies have mainly focused on GLUT1, and sufficient evidence is still lacking regarding the direct deubiquitination mechanisms of other GLUTs and changes in glucose metabolic flux. Future studies should combine ubiquitination site identification, membrane localization analysis, and stable isotope tracing to further clarify the regulatory mechanisms of DUBs in glucose transport and metabolic reprogramming, and to evaluate their potential value in tumor metabolic intervention and immunotherapy.

### 2.3. Deubiquitination of Upstream Regulators of Glycolysis

In addition to directly regulating glycolytic enzymes and glucose transporters, deubiquitinating enzymes (DUBs) can also indirectly affect glycolysis in tumor cells by regulating the ubiquitination status and protein stability of upstream regulators of glycolysis. These upstream regulators mainly include metabolism-related transcription factors, RNA-binding proteins, and signaling kinases.

As an important transcription factor regulating cell proliferation and energy metabolism, c-Myc promotes the expression of multiple glycolysis-related genes, and its protein stability is strictly regulated by the ubiquitin–proteasome system. In pancreatic ductal adenocarcinoma (PDAC), hypoxia and increased extracellular matrix stiffness can induce the expression of ubiquitin-specific protease 7 (USP7), which reduces c-Myc ubiquitination and increases its protein stability, thereby promoting the expression of glycolysis-related genes such as glucose transporter 1 (GLUT1), hexokinase 2 (HK2), and lactate dehydrogenase A (LDHA), and consequently enhancing aerobic glycolysis and tumor growth [13]. Similarly, in prostate cancer PC-3 cells, USP17 can directly remove ubiquitin modifications from c-Myc, inhibit its proteasomal degradation, and increase c-Myc protein stability, thereby promoting HK2 and LDHA expression and enhancing glucose consumption, lactate production, and cell proliferation [42]. In addition to USP7 and USP17, other DUBs can also participate in glycolytic reprogramming in different tumors by regulating c-Myc protein stability. In clear cell renal cell carcinoma (ccRCC), USP3 can interact with MYC and reduce its ubiquitination level, prolong MYC protein half-life, and promote the expression of glycolysis-related genes and metabolic activity, thereby enhancing tumor cell proliferation, migration, and invasion [43]. In bladder cancer (BLCA), USP43 can remove K48-linked polyubiquitin chains from lysine residues 148 and 289 (K148 and K289) of c-Myc, inhibit its proteasomal degradation, and thus enhance the transcriptional regulatory function and glycolytic activity of c-Myc [44]. Taken together, these studies indicate that different DUBs can act on the same metabolic transcription factor in different tumor contexts and maintain the expression of glycolysis-related genes by regulating its ubiquitination level and protein turnover. Given that transcription factors such as c-Myc are difficult to target directly pharmacologically, their upstream DUBs may provide new therapeutic entry points for intervening in tumor glycolysis.

In addition to c-Myc, DUBs can also regulate glycolysis and tumor therapeutic responses by stabilizing other metabolism-related transcription factors. Hypoxia-inducible factor 1α (HIF-1α) is an important transcriptional regulator mediating hypoxia adaptation and glycolytic reprogramming in tumor cells, and its protein stability is dynamically regulated by ubiquitin-dependent degradation. In hepatocellular carcinoma (HCC), USP29 can directly deubiquitinate and stabilize HIF-1α, enhance its transcriptional activity, and thereby upregulate the expression of the downstream target HK2, promoting aerobic glycolysis and mediating sorafenib resistance. Knockdown of USP29 can reduce HIF-1α protein levels and glycolytic activity and restore the sensitivity of sorafenib-resistant HCC cells to the drug in both in vitro and in vivo models [45]. In lung adenocarcinoma (LUAD) harboring wild-type p53, ubiquitin-specific protease 54 (USP54) directly interacts with p53, reduces its ubiquitination level, and inhibits protein degradation, thereby increasing p53 protein stability. Stabilized p53 further downregulates GLUT1 expression, inhibits glucose uptake and aerobic glycolysis, and ultimately attenuates tumor cell proliferation [46]. These studies indicate that DUBs, by regulating the protein stability of different metabolism-related transcription factors, can not only affect the expression of glycolysis-related genes but may also participate in hypoxia adaptation and therapeutic resistance of tumor cells.

In addition to transcriptional regulation, DUBs can also regulate tumor glycolysis at the levels of post-transcriptional processing and post-translational modification by stabilizing RNA-binding proteins and signaling kinases. In estrogen receptor-positive (ER+) breast cancer, USP46 removes K48-linked polyubiquitin chains from the RNA-binding protein polypyrimidine tract-binding protein 1 (PTBP1), inhibits its proteasomal degradation, and increases its protein stability, thereby promoting alternative splicing of pyruvate kinase M (PKM), increasing the PKM2/PKM1 expression ratio, and enhancing glycolytic activity and tamoxifen resistance; knockdown of USP46 reverses the above processes and increases the sensitivity of tumor cells to tamoxifen [47]. Furthermore, in non-small cell lung cancer (NSCLC), USP7 removes K48-linked polyubiquitin chains from the non-receptor tyrosine kinase c-Abl, increases its protein stability, and enhances the interaction between c-Abl and 14-3-3α/β proteins, promoting the accumulation of c-Abl in the cytoplasm. Cytoplasmic c-Abl further promotes phosphorylation of hexokinase 2 (HK2) and reduces its ubiquitination level, increasing the stability and enzymatic activity of HK2, thereby enhancing glycolysis and promoting tumor progression; knockdown of USP7 or c-Abl can inhibit glycolysis and lactate production [48]. These studies indicate that DUBs can not only regulate the expression of glycolysis-related genes by stabilizing transcription factors, but can also indirectly affect the isoform expression, protein stability, and catalytic activity of glycolytic enzymes by regulating the stability of RNA-binding proteins and signaling kinase-mediated post-translational modifications, reflecting the multi-level regulatory roles of DUBs in tumor glucose metabolic reprogramming.

### 2.4. Non-Catalytic Functions of DUBs and Their Potential Roles in the Regulation of Tumor Glycolysis

In addition to their classical deubiquitinating catalytic functions, some deubiquitinating enzymes (DUBs) can also participate in cellular signal transduction through non-catalytic mechanisms, including competitive substrate binding, inhibition of E2-mediated ubiquitin transfer, and modulation of protein–protein interactions, and may thereby affect tumor glycolytic reprogramming. Competitive blockade of E3 ubiquitin ligase–substrate binding is an important mode of non-canonical DUB regulation. In colorectal cancer, OTUB2 impedes the binding of Parkin to PKM2, thereby reducing PKM2 ubiquitination and enhancing its enzymatic activity, thus promoting glycolysis. OTUB2 knockout reduces glucose consumption, lactate production, and ATP levels; however, whether this effect is independent of its catalytic activity remains unclear [49]. In bladder cancer, in addition to stabilizing c-Myc through catalytic deubiquitination, USP43 can also impede E3 ligase FBXW7-mediated protein degradation by competitively binding to c-Myc. The catalytically inactive mutant USP43 C110S still partially maintained c-Myc protein levels, supporting a non-catalytic protective mechanism [44]. Given that c-Myc is an important transcriptional regulator of glycolysis-related genes, this mechanism may participate in glycolytic reprogramming; however, its independent metabolic contribution remains to be defined.

DUBs can also regulate the stability of metabolism-related transcription factors by inhibiting the ubiquitination cascade. Studies have shown that OTUB1 can reduce K48-linked polyubiquitination of hypoxia-inducible factor 1α (HIF-1α) by non-catalytically inhibiting E2-mediated ubiquitin transfer, thereby enhancing its protein stability and transcriptional activity. The catalytically inactive mutant OTUB1 C91A still stabilized HIF-1α and promoted PKM2 transcription, whereas the E2-binding-deficient mutant OTUB1 D88A failed to exert the same effect, indicating that this process mainly depends on the non-catalytic ubiquitination-inhibitory function of OTUB1. Further studies found that OTUB1 deficiency reduced compensatory glycolysis and glucose uptake in lung cancer cells under hypoxic conditions, suggesting that OTUB1 may participate in HIF-1α-mediated glycolytic adaptation through a non-catalytic mechanism [50]. Similarly, USP19 can maintain HIF-1α stability independently of its catalytic activity. Its catalytically inactive mutant USP19 C506S still interacted with HIF-1α and inhibited its degradation, whereas USP19 knockdown attenuated HIF-1α accumulation and the expression of its target gene GLUT1 under hypoxic conditions [51]. These results suggest that USP19 may participate in hypoxic metabolic adaptation through a non-catalytic protein-protective mechanism; however, its direct effect on glycolytic flux has not yet been validated.

DUBs can also affect the function of transcription factors through non-catalytic protein–protein interactions. Studies have shown that, under hypoxic conditions, USP43 enhances its interaction with 14-3-3 proteins (especially 14-3-3ζ and 14-3-3ε) in a serine phosphorylation-dependent manner, promoting the association of 14-3-3 with HIF-1α, thereby enhancing nuclear accumulation of HIF-1α and its binding to hypoxia-response elements (HREs) in target genes and increasing HIF-1-dependent transcriptional activity. This process is independent of increased HIF-1α protein stability, and the catalytically inactive USP43 mutant still partially restored HIF-1 transcriptional responses, supporting a non-catalytic regulatory role. Further studies found that USP43 deficiency affected the expression of glycolysis-related genes under hypoxic conditions and reduced basal glycolytic levels in lung cancer cells after HIF signaling was activated by the PHD inhibitor Roxadustat [52]. These results suggest that USP43 may participate in HIF-1-mediated glycolytic regulation through non-catalytic protein–protein interactions; however, the independent contribution of its non-catalytic function to glycolysis still requires further validation.

In summary, DUBs participate in tumor glycolytic reprogramming at multiple levels—including glycolytic enzyme activity, glucose uptake, and metabolic gene expression—through mechanisms such as direct deubiquitination and non-catalytic regulation. These regulatory modes are interconnected and jointly affect the metabolic adaptation and malignant progression of tumor cells, and they also provide potential therapeutic strategies for targeting DUBs to intervene in tumor glycolysis (Table 1).

## 3. DUBs Indirectly Regulate Glucose Metabolism Through Metabolic Signaling Pathways

DUBs stabilize or activate key metabolic regulatory proteins through deubiquitination, thereby modulating the HIF-1 signaling pathway, the AMPK pathway, and the AKT/mTOR pathway, among others, to influence cellular glucose uptake, utilization, and storage [21]. Notably, these metabolic signaling pathways are not independent of one another but instead jointly regulate cellular energy homeostasis and metabolic adaptation through complex interactions. Therefore, the effects of DUBs on glucose metabolism depend not only on their directly regulated molecular targets but may also be influenced by the activities of other signaling pathways and the cellular metabolic status.

### 3.1. mTOR Pathway

Mammalian target of rapamycin complex 1 (mTORC1) is considered a central hub of cellular metabolic regulation, and its activity directly determines the dynamic balance of multiple metabolic pathways, including glycolysis, the pentose phosphate pathway, and lipid synthesis [54]. Accumulating evidence indicates that characteristic ubiquitination events on mTOR complexes, as well as the regulatory roles of associated E3 ligases and DUBs, play critical roles in maintaining their pathological and physiological functions [55]. Recent studies have gradually revealed multiple mechanisms by which DUBs indirectly influence tumor glucose metabolism through the regulation of the mTOR signaling pathway. Loss of ubiquitin-specific protease 10 (USP10) enhances mTOR/S6K signaling activity in colon cancer cells, thereby promoting HIF-1α protein synthesis without markedly affecting HIF-2α protein levels. Meanwhile, USP10 loss is accompanied by increased extracellular acidification, glycolytic capacity, and glycolytic reserve, and promotes several malignant phenotypes. Further studies showed that the mTOR inhibitor rapamycin attenuates the USP10 loss-induced increase in HIF-1α protein levels, supporting the involvement of mTOR/S6K signaling in this regulatory process [56]. However, the specific upstream molecular mechanism by which USP10 loss enhances mTOR/S6K signaling remains to be elucidated. In non-small cell lung cancer, OTUB2 activates the AKT/mTOR pathway through U2 small nuclear RNA auxiliary factor 2 (U2AF2)-dependent deubiquitination, thereby enhancing the Warburg effect and significantly promoting tumor proliferation and metastasis; its expression level is positively correlated with U2AF2 and phosphoglycerate kinase 1 (PGK1) [57]. Similarly, in breast cancer, upstream stimulatory factor 1 (USF1) transcriptionally activates AT1 receptor-associated protein (ATRAP), which in turn relies on ubiquitin-specific protease 14 (USP14)-mediated stabilization of pre-B-cell leukemia transcription factor 3 (PBX3), ultimately leading to AKT/mTOR signaling activation and driving glycolysis, proliferation, and metastasis, forming a malignant USF1-ATRAP-PBX3 signaling axis [58]. Furthermore, ubiquitin-specific protease 38 (USP38) deubiquitinates and stabilizes monocarboxylate transporter 1 (MCT1) to promote lactate efflux, thereby activating the AKT/mTOR pathway and reducing intracellular lactate accumulation. Although demonstrated in a non-neoplastic PM2.5-induced lung injury model, this study illustrates how DUB-dependent lactate transport can feed into AKT/mTOR signaling, and suggests a potential role for glucose metabolic reprogramming in environmental exposure-related diseases and tumor risk [59]. Overall, DUBs indirectly reshape the cellular glucose metabolic network by modulating the activity of the mTOR signaling pathway. This finding not only reveals the intimate connection between deubiquitination modifications and metabolic reprogramming but also provides a theoretical basis for potential therapeutic targets in related diseases. However, most current studies remain focused on the single-pathway effects of individual DUBs, and their universality across different tumor types as well as their interactions with other metabolic regulatory factors remain unclear. Therefore, future research urgently needs to systematically dissect the network characteristics of the DUBs-mTOR axis in tumor metabolic reprogramming, as well as its functions in drug resistance and immune microenvironment shaping, thereby advancing clinical translation and application.

### 3.2. AMPK Pathway

AMPK is a central sensor of cellular energy homeostasis and plays a pivotal role in the regulation of glucose metabolism. When energy supply is insufficient and the intracellular AMP/ATP ratio increases, AMPK is activated, thereby promoting glycolysis, glucose transport, and fatty acid oxidation while inhibiting gluconeogenesis and lipid synthesis to restore energy balance [60]. Notably, the regulation of tumor glycolysis by AMPK is context-dependent, involving not only the modulation of metabolic enzymes and glucose transport processes but also close crosstalk with mTORC1-mediated protein synthesis and cell growth signaling. This regulatory complexity is also evident in the process of autophagy. As a cellular stress response mechanism closely linked to AMPK–mTORC1 signaling, autophagy can exert either tumor-suppressive or cytoprotective effects depending on the cellular context. For example, following proteasome inhibition, autophagy facilitates the recovery of proliferative capacity in MCF7 breast cancer cells, whereas concomitant inhibition of autophagy prevents this recovery, highlighting the potential therapeutic value of combination strategies [61]. These findings further underscore the importance of considering adaptive survival mechanisms when targeting metabolic regulation in cancer. Deubiquitination plays an important regulatory role in this process. Mechanistically, USP10 removes K63-linked polyubiquitin chains from AMPKα to facilitate LKB1-mediated phosphorylation and full activation of AMPK. Under conditions of USP10 deficiency, insufficient deubiquitination of AMPKα impairs AMPK activation, and the expression of downstream glycolysis-related genes such as ALDOA, lactate dehydrogenase A (LDHA), and pyruvate dehydrogenase kinase 1 (PDK1) is significantly elevated, suggesting that deubiquitination modification of AMPK is crucial for the dynamic regulation of glycolysis [62].

AMPK can further influence cellular metabolic states by modulating mTORC1 signaling. Previous studies have demonstrated that AMPK inhibits mTORC1 activity by phosphorylating tuberous sclerosis complex 2 (TSC2) and Raptor, a component of mTORC1, thereby suppressing anabolic processes associated with protein synthesis and cell growth [63,64]. Given that USP10 promotes AMPK activation and that mTORC1 is involved in regulating HIF-1α protein synthesis, these established molecular interactions suggest that USP10 may indirectly modulate HIF-1α expression and associated glucose metabolic processes through the AMPK–mTORC1 signaling axis. However, direct experimental evidence establishing alterations in AMPK activity as a necessary intermediate step in the enhanced mTOR/S6K signaling observed upon USP10 loss remains lacking. Therefore, this proposed regulatory mechanism warrants further experimental validation.

In addition, in non-tumor models of lysosomal damage and mycobacterial infection, lysosomal membrane damage triggers cytosolic lectin Galectin-9 to recognize exposed glycan chains, which in turn leads to the dissociation of ubiquitin-specific protease 9X (USP9X) from TAK1 and activation of the TAK1 K63-linked ubiquitination cascade, thereby initiating the AMPK–autophagy pathway on damaged lysosomes to maintain endomembrane homeostasis and eliminate pathogens [65]. Furthermore, while this AMPK–autophagy regulatory pathway has a well-defined function in infection defense, it may potentially be involved in tumor metabolic reprogramming [66]. On the one hand, AMPK activation can suppress cancer cells that rely on aerobic glycolysis for rapid proliferation by inhibiting mTORC1 activity and limiting the glycolytic pathway; on the other hand, enhanced AMPK-induced autophagy may provide tumor cells with a metabolic adaptive advantage in hypoxic or nutrient-deprived environments. Therefore, the lysosomal damage sensing-AMPK-autophagy pathway may exhibit a “double-edged sword” effect in tumor initiation and progression: it possesses metabolic tumor-suppressive potential, yet may also promote tumor survival and progression under specific conditions. In summary, DUBs play an indirect but important role in maintaining glucose metabolic homeostasis and reprogramming through fine regulation of upstream and downstream molecules of the AMPK signaling axis. This understanding not only expands our knowledge of ubiquitination/deubiquitination modifications in metabolic homeostasis but also provides potential novel therapeutic targets for the intervention of diseases such as type 2 diabetes, obesity, and tumor metabolic reprogramming. Future studies need to further elucidate the specific roles of different DUBs in AMPK regulation and their causal relationship with cancer metabolic adaptation, in order to explore their feasibility as precision medicine targets.

### 3.3. HIF-1 Signaling Pathway

Hypoxia-inducible factor 1 (HIF-1) is a core transcription factor of the glycolytic pathway. By upregulating the expression of glucose transporters (GLUTs) and multiple key glycolytic enzymes, it enables tumor cells to maintain energy supply and achieve metabolic adaptation under hypoxic conditions [67]. Accumulating evidence indicates that multiple DUBs can drive tumor glycolytic reprogramming by directly or indirectly regulating the stability and transcriptional activity of HIF-1α. For example, ubiquitin-specific protease 25 (USP25) maintains glycolytic flux and tumor growth in pancreatic ductal adenocarcinoma (PDAC) by stabilizing HIF-1α, and targeting USP25 can selectively kill tumor cells and significantly suppress PDAC progression [19]. USP43, on the other hand, recruits 14-3-3 proteins to bind HIF-1α through hypoxia-induced phosphorylation events, thereby promoting its nuclear accumulation and enhancing HIF-1 transcriptional responses. Notably, this function is independent of its deubiquitinase activity, suggesting that DUBs may also regulate signaling through scaffolding roles beyond their classical enzymatic functions [52].

In addition to directly regulating the protein stability or transcriptional activity of HIF-1α, DUBs can also indirectly modulate HIF-1α protein levels by affecting upstream metabolic signaling pathways. For example, the aforementioned studies showed that USP10 deficiency promotes HIF-1α protein synthesis by enhancing mTOR/S6K signaling, rather than by altering its transcriptional level or protein degradation rate [56]. This finding suggests that DUB-mediated regulation of HIF-1α is not limited to the classical ubiquitination-dependent protein degradation process but may also involve the regulation of protein synthesis. However, the specific molecular link between USP10 and mTOR/S6K remains unclear, and whether it is influenced by other metabolic signaling pathways, such as AMPK, requires further investigation.

USP21 indirectly elevates HIF-1α levels by stabilizing heat shock protein 90 (HSP90), thereby upregulating the transcription of key glycolytic genes, including enolase 2 (ENO2), enolase 3 (ENO3), aldolase C (ALDOC), and acyl-CoA synthetase short chain family member 2 (ACSS2). This enhances aerobic glycolysis and supports the proliferation of cholangiocarcinoma (CCA) cells [68]. Furthermore, USP28 deubiquitinates PKM2 to enhance PKM2-dependent HIF-1α signaling and glycolytic gene expression, thereby promoting glycolysis and cholangiocarcinoma progression; USP29, in turn, directly removes ubiquitin chains from HIF-1α to prevent its proteasomal degradation, thereby sustaining its activation under hypoxic conditions [45,69]. Notably, in sorafenib-resistant hepatocellular carcinoma (HCC), HIF-1α—but not HIF-2α—is significantly upregulated, and its depletion reverses drug resistance and induces cell death, suggesting that HIF-1α serves as a critical driver of drug tolerance. However, the precise regulatory mechanisms underlying its function in resistant cells and in patients remain to be further elucidated [45]. Similarly, ubiquitin-specific protease 11 (USP11) promotes proliferation and metastasis of HCC cells by stabilizing HIF-1α and activating pyruvate dehydrogenase kinase 1 (PDK1)/LDHA-driven glycolysis, providing a novel theoretical basis for metabolic intervention therapy [70]. OTU deubiquitinase, ubiquitin aldehyde binding 1 (OTUB1), stabilizes HIF-1α through its non-canonical ubiquitination-inhibitory activity rather than classical substrate deubiquitination. This regulatory effect operates independently of the canonical PHD/VHL degradation pathway, further enhancing HIF-1α transcriptional activity and glycolytic reprogramming and thereby promoting tumor hypoxic adaptation [50]. Ubiquitin-specific protease 22 (USP22), under hypoxic conditions, maintains stemness and chemoresistance in nasopharyngeal carcinoma cells by stabilizing HIF-1α, and this effect is dependent on the glycolytic pathway, suggesting that targeting USP22 may represent a novel strategy to overcome hypoxia-associated drug resistance [71]. In summary, DUBs regulate HIF-1α and the glycolytic network through diverse mechanisms, acting either directly via deubiquitination to stabilize HIF-1α, or indirectly by modulating chaperone proteins or signaling complexes to enhance its activity. These studies not only reveal the multifaceted roles of DUBs in hypoxic adaptation and metabolic reprogramming, but also suggest that they may represent important entry points for metabolic targeted therapy and intervention against drug resistance. Nevertheless, the differential regulatory modes of distinct DUBs toward HIF-1α, as well as their tumor type-specific effects, warrant further systematic investigation to explore therapeutically actionable windows that can be precisely leveraged.

### 3.4. Other Signaling Pathways

Beyond classical pathways such as PI3K/AKT and mTOR, accumulating evidence indicates that DUBs participate in tumor glycolytic reprogramming by regulating distinct signaling axes. UCHL5 is highly expressed in HCC and is significantly correlated with poor prognosis. Mechanistically, ubiquitin carboxyl-terminal hydrolase L5 (UCHL5) binds to β-catenin to inhibit its ubiquitin-mediated degradation, thereby activating the Wnt/β-catenin signaling pathway, which in turn enhances glycolysis and drives proliferation and metastasis of HCC cells. Thus, UCHL5 not only serves as a critical driver of HCC progression but may also represent a novel prognostic biomarker and therapeutic target [72]. Similarly, ubiquitin-specific protease 24 (USP24) is highly expressed in gastric cancer and is closely associated with poor prognosis. It promotes cancer cell proliferation and glycolysis by stabilizing Polo-like kinase 1 (PLK1) and activating the Notch1 signaling pathway, whereas USP24 silencing reverses these effects, suggesting that the USP24/PLK1/Notch1 axis may represent a critical entry point for metabolic intervention in gastric cancer [73]. Notably, not all DUBs exhibit pro-tumorigenic effects. For instance, overexpression of ubiquitin-specific protease 13 (USP13) can significantly inhibit tumor cell proliferation, invasion, and glycolysis by upregulating phosphatase and tensin homolog (PTEN) and blocking the AKT signaling pathway, suggesting that different DUBs may exert opposing regulatory roles in metabolic reprogramming [74]. In addition, WD repeat domain 1 (WDR1) inhibits ubiquitin-mediated degradation of β-catenin by binding to USP7, thereby upregulating c-Myc expression, suggesting that USP7 and the Wnt/β-catenin signaling exert synergistic effects to collectively drive glycolysis in PDAC [13]. In breast cancer, angiotensin II type 1 receptor-associated protein (ATRAP) is transcriptionally activated by USF1 and stabilizes PBX3 via USP14, ultimately activating the AKT/mTOR pathway to promote breast cancer cell growth, metastasis, and glycolysis. Its expression level is positively correlated with poor prognosis, constituting a targetable USF1/ATRAP/PBX3 signaling axis [58]. Furthermore, OTUB2 drives glycolysis, proliferation, and invasion in endometrial cancer by regulating the PKM2/PI3K/AKT signaling pathway. Its overexpression exerts pro-tumorigenic effects, whereas its silencing exhibits tumor-suppressive effects, suggesting that OTUB2 may serve as a potential prognostic biomarker and therapeutic target in this malignancy [75]. Finally, USP21 promotes cellular glycolysis and proliferation through regulating the STAT3/FOXO1 signaling axis. The metabolic and proliferative defects caused by USP21 knockdown can be rescued by reactivation of this pathway, whereas USP21 overexpression further accelerates in vivo tumor growth [76]. Collectively, these studies demonstrate that DUBs can regulate tumor glucose metabolism through crosstalk with multiple signaling pathways, and exhibit context-dependent pro-tumorigenic or tumor-suppressive effects across different cancer types. This not only reveals the complexity of DUBs in metabolic reprogramming, but also highlights their potential value as prognostic indicators and precision therapeutic targets. However, most existing studies have focused on individual tumor types and single signaling axes, and the universality and specificity of these mechanisms across diverse tumor contexts warrant further systematic investigation.

Collectively, DUBs regulate glucose metabolism through interconnected signaling pathways, including mTOR, AMPK, HIF-1, and other metabolic regulatory networks (Table 2). Their effects are highly context-dependent and may either promote or suppress glycolysis depending on the tumor type and molecular targets. Further studies are needed to clarify the crosstalk among these pathways and evaluate the therapeutic potential of targeting DUB-mediated metabolic reprogramming.

## 4. The Bridging Role of DUBs in the Interplay Between Tumor Immune Microenvironment and Metabolism

**Figure 2:**
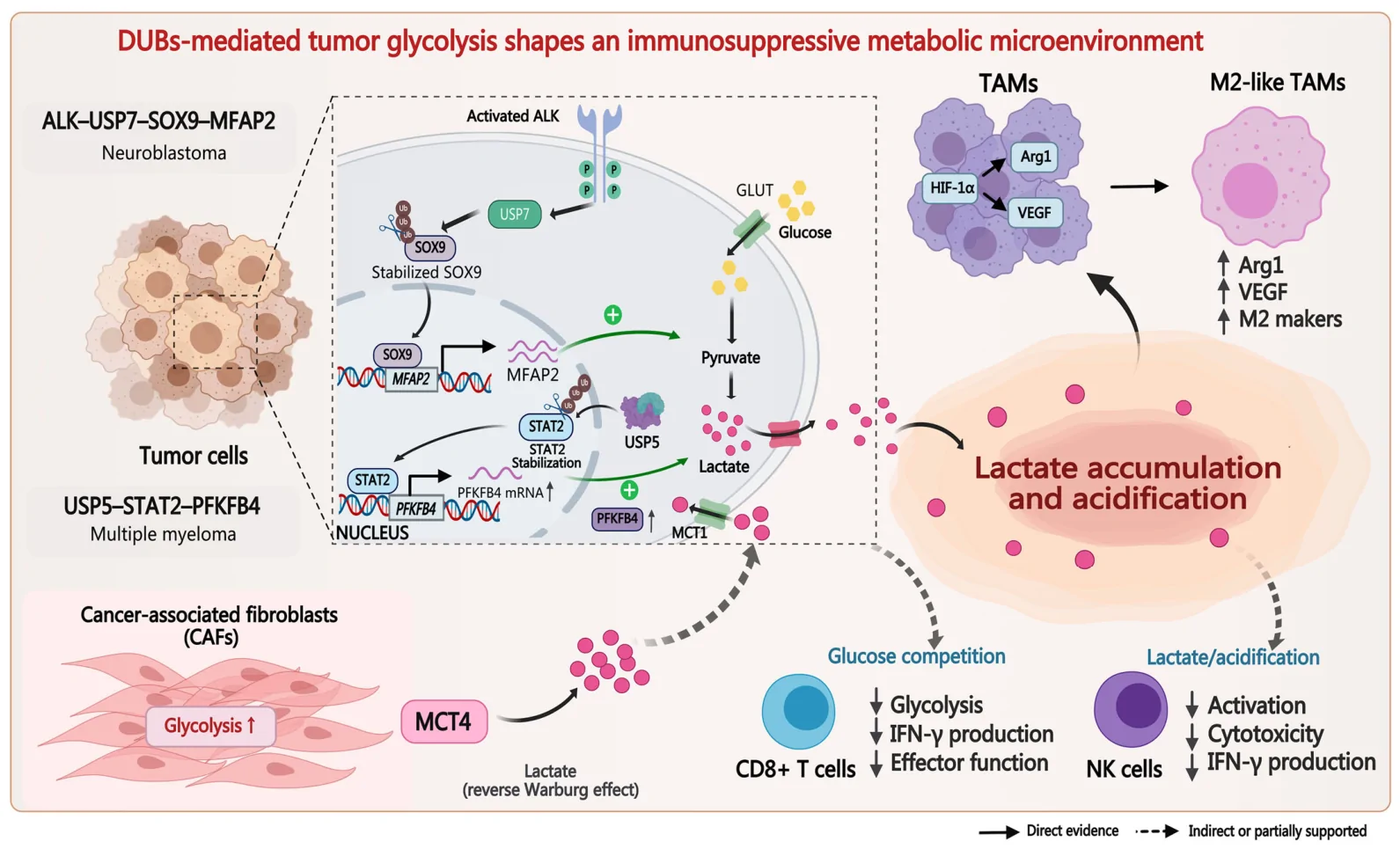
DUB-mediated tumor glycolytic reprogramming and its potential impact on the tumor immune microenvironment. DUBs regulate tumor glycolysis through distinct molecular pathways, including the USP5–STAT2–PFKFB4 and ALK–USP7–SOX9–MFAP2 axes, thereby promoting glucose metabolism and lactate production in their respective tumor contexts. Lactate accumulation and extracellular acidification may contribute to an immunosuppressive microenvironment by favoring M2-like tumor-associated macrophage (TAM) polarization and impairing the effector functions of CD8^+^ T cells and natural killer (NK) cells. Cancer-associated fibroblasts (CAFs) may also contribute to extracellular lactate accumulation through glycolysis and monocarboxylate transporter 4 (MCT4)-mediated lactate export. Solid arrows indicate relationships supported by experimental evidence in the respective models, whereas dashed arrows represent indirect or incompletely validated immunometabolic interactions. The integration of these pathways does not necessarily imply that all proposed connections have been directly demonstrated within the same tumor model. Created with MedPeer (medpeer.cn). Certificate No.: f9da29u63sme34x8c1792670804.

DUBs play an important bridging role between tumor metabolic reprogramming and immune microenvironment remodeling by regulating glycolysis in tumor cells, immune cell metabolism, and immune checkpoint signaling (Figure 2).

### 4.1. DUB-Mediated Reprogramming of Tumor Glycolysis Shapes an Immunosuppressive Metabolic Microenvironment

Recent studies have revealed that DUBs can influence the phenotype of tumor-associated macrophages (TAMs) by regulating glycolysis-related signaling axes. In multiple myeloma, USP5 reduces STAT2 ubiquitination and increases its protein stability, thereby enhancing PFKFB4 transcription, promoting glycolysis and lactate production, and consequently increasing the ability of tumor cells to induce macrophage polarization toward an M2-like phenotype. USP5 knockdown, STAT2 functional intervention, and lactate dehydrogenase inhibition experiments further supported the role of this signaling axis, and animal experiments also showed that USP5 knockdown was accompanied by reduced tumor growth and decreased M2-like macrophage-associated markers [77]. Similarly, in neuroblastoma, ALK recruits and activates USP7, promoting deubiquitination-mediated stabilization of SOX9 and transcriptional expression of MFAP2, thereby enhancing tumor glycolysis, lactate production, and M2-like macrophage-associated phenotypes; the ALK inhibitor lorlatinib inhibited these changes, whereas MFAP2 overexpression partially reversed its antitumor effects [78]. Although these two signaling axes involve different DUBs and downstream molecules, both exhibit common features of enhanced glycolysis, increased lactate production, and enhanced M2-like macrophage-associated phenotypes, suggesting that DUB-mediated metabolic reprogramming may participate in the establishment of an immunosuppressive microenvironment. Notably, lactate in the tumor microenvironment is not solely derived from tumor cells. In addition to tumor cells, cancer-associated fibroblasts (CAFs) also participate in metabolic reprogramming within the tumor microenvironment. In some tumor models, CAFs exhibit enhanced glycolytic activity and release lactate extracellularly via monocarboxylate transporter 4 (MCT4), whereas adjacent tumor cells can take up lactate via monocarboxylate transporter 1 (MCT1) and use it for mitochondrial oxidative metabolism. This intercellular metabolic cooperation is considered a manifestation of the reverse Warburg effect [79,80]. These studies indicate that lactate metabolism in the tumor microenvironment is influenced not only by the glycolytic activity of tumor cells themselves but also by metabolite exchange between stromal cells and tumor cells.

In this context of multicellular metabolic interactions, DUB-driven enhancement of tumor glycolysis may also affect other immune cells through metabolic competition. On the one hand, increased glucose consumption can limit glycolysis and IFN-γ production by CD8^+^ cytotoxic T lymphocytes (CTLs); on the other hand, lactate accumulation and local acidification can inhibit the activation and effector functions of CTLs and natural killer (NK) cells [81,82]. Melanoma studies have shown that LDHA-driven lactate production can suppress IFN-γ production by affecting NFAT expression, whereas reducing the lactate-producing capacity of tumors can increase the infiltration of IFN-γ-positive T cells and NK cells [82]. Therefore, the glycolysis-promoting effects of DUBs such as USP25, USP11, USP5, and USP7 may indirectly attenuate antitumor immunity by enhancing glucose competition and lactate-related metabolic stress; however, direct mechanistic validation of this link is still lacking.

Beyond metabolic suppression, lactate can also act as a signaling molecule to promote the expression of Arg1 and VEGF in TAMs through HIF-1α-dependent mechanisms and may regulate macrophage transcriptional programs through histone lactylation. Related studies have found that restricting lactate-derived carbon from entering mitochondrial metabolism does not necessarily block lactate-associated macrophage polarization, suggesting that its effects may involve metabolic signaling and epigenetic regulation rather than substrate utilization alone [83]. Accordingly, DUB-promoted lactate accumulation may participate in functional remodeling of TAMs through HIF-1α signaling and histone lactylation. However, whether the USP5/STAT2/PFKFB4 and USP7/SOX9/MFAP2 signaling axes directly regulate TAM polarization through specific lactylation events has not yet been demonstrated. Future studies integrating immunocompetent models, cell-specific interventions, and metabolic tracing are still needed to clarify the mechanisms of DUB-mediated metabolic–immune crosstalk and their tumor type specificity.

### 4.2. DUBs Remodel Antitumor Immune Function by Regulating Immune Cell Metabolism

Glucose competition, lactate accumulation, and nutrient restriction within the tumor microenvironment (TME) can reshape the metabolic status and effector functions of infiltrating immune cells. Deubiquitinating enzymes (DUBs) participate in the metabolic adaptation of distinct immune cells by regulating metabolic signaling, protein translation, and mitochondrial homeostasis, with their effects exhibiting marked cell-type dependency. In CD8^+^ T cells, OTUB1 restricts antitumor activity by inhibiting AKT-dependent glycolysis. Specifically, OTUB1 limits IL-15-mediated T cell priming by suppressing K63-linked ubiquitination and membrane localization of AKT. OTUB1 deficiency enhances GLUT1 and HK2 expression and glycolytic capacity, while increasing effector cytokine production and antitumor activity; these metabolic and functional alterations are reversed by AKT inhibition [84]. Furthermore, the lactate-induced USP7–CMC1 axis promotes terminal differentiation and exhaustion of CD8^+^ T cells, whereas chronic T cell receptor stimulation-induced USP30 exacerbates dysfunction by inhibiting mitophagy. Inhibition of CMC1 or USP30 helps restore antitumor immunity; however, these mechanisms primarily involve mitochondrial homeostasis, and direct evidence for the regulation of glycolytic flux remains lacking [85,86]. In regulatory T cells (Tregs), DUBs maintain immunosuppressive functions by restricting aberrant glycolysis. USP47 removes K63-linked ubiquitin chains from YTHDF1, thereby inhibiting YTHDF1-mediated c-Myc mRNA translation and limiting excessive glycolysis. USP47 deficiency results in enhanced glycolysis, decreased Foxp3 expression and immunosuppressive function, and suppressed tumor growth [87]. Additionally, USP22 and USP21 support the functional adaptation of Tregs under glucose restriction and hypoxic conditions by stabilizing Foxp3; however, sufficient evidence is lacking to demonstrate that they directly regulate glycolysis [88]. In tumor-associated macrophages (TAMs), DUBs influence the immunosuppressive phenotype by modulating metabolic pathway selection. USP14 promotes PGC-1α-related fatty acid oxidation by stabilizing SIRT1, thereby supporting M2-like polarization; conversely, breast cancer-derived exosomal circ-0100519 promotes immunosuppressive macrophage polarization via the USP7–NRF2 axis [89,90]. These studies primarily involve fatty acid metabolism and redox adaptation, and their direct impact on glycolysis remains to be elucidated. In summary, DUBs do not unidirectionally promote or suppress glycolysis; rather, they coordinate the metabolic demands and functional states of distinct immune cells through substrate-specific regulation. While OTUB1-mediated restriction of glycolysis impairs CD8^+^ T cell effector functions, USP47-mediated suppression of excessive glycolysis helps maintain the immunosuppressive activity of Tregs. This cell-type dependency suggests that targeting DUBs for tumor immunotherapy should fully consider the metabolic requirements of different immune cells and the potential off-target immune effects.

### 4.3. DUBs Coordinate Metabolic Adaptation and Immune Checkpoint Signaling to Promote Tumor Immune Evasion

Tumor metabolic adaptation and immune checkpoint regulation are interconnected through shared signaling networks and post-translational modifications. DUBs participate in glycolytic reprogramming and immune evasion by stabilizing metabolic regulators or directly modulating PD-L1 degradation. In non-small cell lung cancer, acetate metabolism enhances the binding of USP10 to c-Myc via DLAT-mediated K148 acetylation of c-Myc, thereby promoting its deubiquitination and stabilization. This subsequently upregulates LDHA, MCT1, and PD-L1, concurrently enhancing glycolysis and immune evasion; blocking acetate uptake improves the efficacy of anti-PD-1 therapy [91]. In hepatocellular carcinoma, TKTL1 recruits USP9X to stabilize HIF-1α, promoting HK2-dependent glycolysis and lactate production, and influencing dendritic cell (DC) recruitment via CCL4. Lactate further induces K408 lactylation of TRIM28 in DCs, thereby reducing the ubiquitination and degradation of PD-L1 and forming a transcellular metabolic–immune regulatory network. Notably, high TKTL1 expression is associated with poor prognosis and increased sensitivity to PD-1 blockade in specific cohorts, suggesting that the therapeutic implications of this network are context-dependent [92].

In conclusion, DUBs participate in tumor immunosuppression and immune evasion through multi-level regulation of the crosstalk between glycolysis and immune signaling, thereby providing potential targets for combination strategies integrating metabolic intervention and immune checkpoint blockade.

## 5. Emerging Metabolism-to-DUB Feedback Mechanisms

Tumor glycolytic reprogramming is not only regulated by DUBs; glucose metabolites and their derived signals can also reciprocally affect DUB expression, catalytic activity, and substrate interactions, forming a bidirectional relationship between metabolic status and deubiquitination regulation.

Lactate can regulate DUB expression through histone lysine lactylation (Kla). In endometrial cancer, lactate accumulation promotes H3K18la enrichment in the USP39 promoter region, upregulating USP39 expression and thereby increasing the protein stability of phosphoglycerate kinase 1 (PGK1); this is associated with activation of PI3K/AKT/HIF-1α signaling and enhanced glycolysis, suggesting a potential positive feedback mechanism in glycolysis. However, because the canonical deubiquitinating catalytic activity of USP39 remains controversial, this study cannot directly demonstrate that lactate enhances the catalytic activity of USP39 [93,94]. In glioblastoma stem cells, lactate similarly promotes USP4 transcription via H3K18la, thereby regulating K48- and K63-linked ubiquitination of ANXA2 and contributing to stem cell maintenance and radiotherapy resistance; however, it has not been demonstrated that USP4 reciprocally promotes lactate production [95]. In addition, lactylomic analysis of hepatocellular carcinoma identified lactylation of USP14, with K336 as a possible regulatory site; however, direct evidence for its effects on USP14 catalytic activity and metabolic function is still lacking [96]. Glucose metabolism can also generate UDP-GlcNAc via the hexosamine biosynthetic pathway (HBP), contributing to the regulation of DUBs by O-GlcNAcylation. Thr203 and Ser214 of USP16 can undergo O-GlcNAc modification; Thr203 is adjacent to the catalytic residue Cys204, and mutation of Thr203 markedly attenuates the deubiquitinating capacity of USP16 toward H2AK119ub. This modification also affects Ser552 phosphorylation, subcellular localization, and PLK1 deubiquitination by USP16, suggesting that glucose metabolism-derived modifications can regulate DUB function by affecting regions adjacent to the catalytic center and crosstalk between modifications; however, the causal relationship with altered tumor glycolysis remains to be further clarified [97]. Furthermore, in non-small cell lung cancer, high glucose levels promote CARM1-mediated methylation of OGT at R348, enhancing OGT–USP9X binding, increasing OGT stability and cellular O-GlcNAcylation levels, and consequently promoting c-Myc expression, glycolysis, and cell proliferation [98]. Changes in energy status caused by glucose metabolism can also directly regulate DUB catalytic activity. Energy stress such as glucose deprivation induces AMPK-mediated phosphorylation of USP10 at Ser76 and enhances its deubiquitinating activity. Activated USP10 removes inhibitory ubiquitin modifications from AMPKα, further promoting AMPK activation and forming a positive feedback loop adaptive to energy stress [62]. In contrast, glucose restriction can also affect protein stability by altering DUB–substrate interactions. For example, glucose deprivation weakens USP7–SIRT7 binding and increases SIRT7 ubiquitination, but this cannot be inferred to reflect a decrease in overall USP7 catalytic activity; in colorectal cancer, glucose starvation enhances the protective effect of OTUB2 on PKM2 by impeding Parkin–PKM2 binding and reducing PKM2 ubiquitination, thereby maintaining PKM2 activity and glycolytic adaptation [49,99].

In summary, existing studies indicate that glucose metabolism-related conditions can affect DUB expression and function through distinct mechanisms, including lactylation-mediated transcriptional regulation, O-GlcNAc modification, AMPK-dependent phosphorylation, and altered substrate interactions. Some studies suggest that metabolism-related feedback regulation may exist under specific conditions; however, these mechanisms are highly context-dependent at the molecular and cellular levels and are insufficient to establish a general bidirectional glycolysis–DUB catalytic activity feedback model. Future studies integrating metabolic flux analysis, DUB activity assays, and site-specific functional validation are still needed to further clarify the relevant causal mechanisms and their therapeutic implications.

## 6. Regulation of Tumor Metabolic Reprogramming and the Warburg Effect by DUB Inhibitors

Compared with direct inhibition of a single glycolytic enzyme, targeting DUBs is expected to achieve multi-level intervention in the tumor glycolytic network by regulating upstream transcription factors, metabolic enzymes, and related signaling pathways (Table 3). To date, inhibitors targeting DUBs such as USP7, USP1, USP25, and USP14 have shown metabolic intervention potential in different tumor models.

In PDAC, USP7 stabilizes c-Myc through deubiquitination and promotes the expression of glycolysis-related genes. The USP7 inhibitor P5091 reduces glucose uptake, lactate production, and ECAR in tumor cells, downregulates GLUT1, HK2, and LDHA expression, and inhibits tumor progression in xenograft models and KPC spontaneous pancreatic cancer mouse models, suggesting that the USP7/c-Myc axis is a potential target for intervening in the Warburg effect in pancreatic cancer [13]. Notably, the antitumor effects of USP7 inhibitors are not limited to metabolic regulation. Previous studies have shown that USP7 inhibition can promote MDM2 degradation, thereby enhancing p53 protein stability and downstream antitumor signaling. However, activation of the MDM2–p53 pathway and the resulting proliferation inhibition or apoptosis do not directly demonstrate glycolysis inhibition. Therefore, the metabolic regulatory effects of USP7 inhibitors need to be distinguished from other antitumor mechanisms according to the molecular background of the tumor and specific metabolic experimental results [100]. In addition to regulating metabolic transcription factors, DUB inhibitors can also modulate tumor metabolism by affecting glycolysis-related proteins and mitochondrial dynamics. In T-cell acute lymphoblastic leukemia (T-ALL), USP1 stabilizes PLK1 through deubiquitination, promoting LDHA expression and lactate production; the USP1 inhibitor ML323 reduces glycolytic activity and cell viability and improves the survival of tumor-bearing mice [101]. In hepatocellular carcinoma, USP1 stabilizes CDK5 to promote Drp1 phosphorylation at Ser616 and mitochondrial fission, and both ML323 treatment and USP1 silencing reduce glucose uptake, suggesting that USP1 also participates in the coordinated regulation of glycolysis and mitochondrial dynamics [102]. In addition, targeting DUB-mediated hypoxic adaptation and the stability of key glycolytic enzymes represents another important approach for inhibiting the Warburg effect. In pancreatic cancer, USP25 directly deubiquitinates and stabilizes HIF-1α, maintaining glycolytic activity under hypoxia and the flux of glucose-derived carbon toward serine and glycine synthesis pathways. USP25 silencing inhibits these metabolic processes, whereas FT206, a dual USP25/USP28 inhibitor, reduces HIF-1α target gene expression, inhibits the growth of patient-derived organoids, and promotes apoptosis in hypoxic regions of xenografts [19]. In oral squamous cell carcinoma, USP14 promotes glycolysis by stabilizing the rate-limiting glycolytic enzyme PFKL; b-AP15, which has USP14/UCHL5 inhibitory activity, increases PFKL ubiquitination and reduces its protein level, and the protective effect of the proteasome inhibitor MG132 further supports the involvement of proteasome-dependent degradation [103]. In bevacizumab-resistant ovarian cancer cells, USP7 promotes glycolysis by stabilizing PTK2; the USP7 inhibitor P22077 reduces glucose consumption and lactate production and enhances cell sensitivity to bevacizumab, although the causal relationship between glycolysis inhibition and drug sensitization still requires further validation [104]. These studies indicate that DUB inhibitors can intervene in tumor metabolic reprogramming through multiple mechanisms and may enhance antitumor therapeutic efficacy by affecting metabolism-related drug resistance mechanisms.

Although DUB inhibitors show certain therapeutic potential, their clinical translation still faces challenges such as functional heterogeneity of targets, insufficient inhibitor selectivity, and potential toxicity. On the one hand, the metabolic regulatory effects of DUBs are markedly context-dependent; for example, USP13 can limit AKT signaling activation and glycolysis by stabilizing PTEN, suggesting that non-selective DUB inhibition may produce unfavorable effects. On the other hand, some DUB inhibitors have multi-target activity and may simultaneously affect protein homeostasis and cellular stress, increasing the difficulty of attributing metabolic effects [39]. The proteasome-related DUB inhibitor VLX1570 was terminated in clinical development due to severe pulmonary toxicity, further highlighting the importance of target selectivity and a safe therapeutic window [105]. Currently, although some USP1 inhibitors have entered early-stage clinical studies, their development has mainly focused on tumor vulnerabilities related to DNA damage repair, and direct evidence that therapeutic benefit can be achieved by inhibiting the Warburg effect in patients is still lacking. It should be noted that decreases in glucose uptake, lactate production, or ECAR observed after DUB inhibitor treatment do not necessarily represent specific inhibition of glycolysis. Because some inhibitors simultaneously affect cell proliferation, protein homeostasis, and cell survival, the associated metabolic changes may also be secondary consequences of cellular stress or death. Therefore, future studies should incorporate cell viability correction, early time-point measurements, stable isotope tracing, and genetic rescue experiments to further clarify the causal relationship between DUB inhibition and glycolytic alterations.

**Table 3:** Roles of DUB inhibitors in regulating tumor glycolytic reprogramming and progress in their development.

Inhibitor	Direct Target and Selectivity	Validated Experimental Models and Major Effects	Glycolysis Evidence	Development Stage	References
P5091	USP7, USP47; limited selectivity	Pancreatic cancer cells, xenografts, and KPC mice; inhibits tumor growth	Reduces glucose uptake, lactate production, and ECAR; downregulates GLUT1, HK2, and LDHA; involves the USP7/c-Myc axis	Preclinical	[13]
P22077	USP7, USP47; dual-target inhibition	Bevacizumab-resistant ovarian cancer cells; enhances drug sensitivity	Reduces glucose consumption and lactate production; involves the USP7/PTK2 axis	Preclinical	[104]
ML323	USP1-UAF1; selective non-competitive inhibition	T-ALL cells and tumor-bearing mice, and liver cancer cells; reduces leukemia cell viability, prolongs survival of tumor-bearing mice, and inhibits mitochondrial fission	Reduces lactate production and glucose uptake; involves the PLK1/LDHA and CDK5/Drp1 axes	Preclinical	[101,102]
FT206	USP25, USP28; dual-target inhibition	Patient-derived pancreatic cancer organoids and xenografts; inhibits growth and promotes apoptosis	Drug treatment downregulates HIF-1α target genes; USP25 silencing reduces serine and glycine synthesis	Preclinical	[19]
FT671	Highly selective USP7 inhibitor; validated in screening systems composed of multiple DUBs as a research tool/preclinical study	Multiple myeloma cells and MM.1S xenografts; promotes MDM2 instability, enhances p53 signaling, and inhibits tumor growth	No direct glycolysis evidence established: high selectivity for MDM2–p53-related antitumor effects has been validated, but the cited studies do not directly demonstrate decreased glycolytic flux or that inhibition of the Warburg effect is the primary therapeutic mechanism	Selective research tool/preclinical research	[100]
b-AP15	USP14, UCHL5; multi-target activity	Oral squamous cell carcinoma cells; promotes PFKL degradation and suppresses pro-tumor phenotypes	Increases PFKL ubiquitination and reduces its protein levels, indirectly supporting the inhibition of glycolysis	Preclinical	[103]
VLX1570	USP14, UCHL5-related activity; limited selectivity	Patients with relapsed/refractory multiple myeloma; severe pulmonary toxicity occurred	Direct glycolysis evidence is still lacking	Phase I clinical trial terminated (NCT02372240)	[105]
KSQ-4279	USP1; highly selective allosteric inhibition	Homologous recombination repair-deficient (HRD) tumor models; enhances the efficacy of PARP inhibitors	Direct glycolysis evidence is still lacking	Phase I clinical trial completed (NCT05240898)	[106]
TNG348	USP1; selective allosteric inhibition	BRCA-mutant and homologous recombination repair-deficient (HRD) tumor models; exhibits antitumor activity	Direct glycolysis evidence is still lacking	Phase I/II clinical trial terminated (NCT06065059)	[107]

## 7. Conclusions

DUBs participate in tumor glycolytic reprogramming by regulating the stability, activity, and subcellular localization of key glycolytic enzymes, glucose transporters, and metabolic regulatory factors, and by affecting signaling pathways such as AMPK, mTOR, HIF-1, and PI3K/AKT. Their effects involve both catalytic deubiquitination and non-catalytic protein–protein interactions and regulation of ubiquitination. Because different DUBs may share substrates and a single DUB can act on different substrates and ubiquitin chain types, their metabolic effects are markedly mechanism- and tumor context-dependent rather than universally promoting glycolysis. The metabolic regulation by DUBs is also closely associated with the tumor immune microenvironment. Some DUBs participate in the formation of an immunosuppressive microenvironment by promoting tumor glycolysis and lactate production, while also directly affecting immune cell metabolism. For example, OTUB1 restricts AKT-dependent glycolysis and effector functions of CD8^+^ T cells, whereas USP47 maintains the immunosuppressive capacity of regulatory T cells by limiting their excessive glycolysis, suggesting cell type-specific roles of DUBs. However, whether changes in tumor glycolysis directly affect immune cell function through glucose competition, lactate signaling, or metabolite exchange still lacks sufficient causal validation, and thus the overall impact of DUBs on antitumor immunity cannot be inferred on this basis. On the other hand, metabolic status can reciprocally affect DUBs through lactylation, O-GlcNAcylation, phosphorylation, and altered substrate interactions. Some studies suggest that metabolites can promote DUB expression and enhance glycolysis, and the mutual regulation between USP10 and AMPK may participate in maintaining metabolic homeostasis. However, these mechanisms have not all been demonstrated to directly alter DUB catalytic activity or glycolytic flux, and they are insufficient to establish a generally applicable bidirectional glycolysis–DUB feedback model. Despite progress, current evidence remains largely focused on individual DUBs and their substrates, and often uses metabolic protein expression, glucose uptake, lactate production, or extracellular acidification rate as glycolytic indicators, lacking systematic validation of actual metabolic flux and causal relationships. Future studies should integrate stable isotope tracing, metabolomics, ubiquitination modification analysis, and DUB activity assays, and employ site-directed mutagenesis, catalytically inactive mutants, and genetic rescue experiments to clarify substrate dependence and catalytic versus non-catalytic mechanisms. Meanwhile, using immunocompetent tumor models, cell-specific interventions, and spatial multi-omics technologies, the metabolic roles of DUBs in tumor cells and different immune cells and their interconnections should be dissected, and validation of metabolic feedback-related modification sites and functions should be strengthened. In terms of therapeutic translation, pharmacological interventions targeting DUBs such as USP7, USP1, USP25, and USP14 have shown metabolic regulatory and antitumor potential in some preclinical models, but multi-target drug activity and secondary metabolic alterations may confound mechanistic attribution. Some DUB inhibitors that have entered clinical studies act mainly through non-metabolic mechanisms, and their efficacy cannot yet be attributed to glycolysis inhibition. Future efforts should improve drug selectivity, define safe therapeutic windows and metabolic mechanisms of action, and identify benefiting populations based on tumor molecular background, substrate dependence, and metabolic phenotype, while evaluating the feasibility of combination with metabolic targeting or immunotherapy. Overall, the mechanisms by which DUBs participate in tumor glycolytic reprogramming are multi-layered and context-dependent. Future research should shift from single-molecule associations to systematic validation of metabolic flux, cell type specificity, and causal mechanisms, so as to clarify their biological significance and therapeutic translational value.

## Figures and Tables

**Table 1:** Molecular mechanisms and evidence supporting the regulation of tumor glycolysis by deubiquitinating enzymes.

DUB	Substrate	Ubiquitin Modification Type and Regulatory Mechanism	Effect on Metabolism	Tumor Experimental Model	Reference
USP3	c-Myc	Reduces c-Myc ubiquitination and prolongs its protein half-life	Enhances c-Myc stability, promotes the expression of glycolysis-related genes, and increases glucose uptake and lactate production	ccRCC; human ccRCC cell lines and tumor tissues	[43]
USP4	PKM2	Inhibits PKM2 protein degradation through deubiquitination, increasing PKM2 protein stability	Increases glucose uptake and lactate production and promotes aerobic glycolysis	GC; AGS and HGC-27 cells	[27]
USP4	LDHA	Inhibits proteasomal degradation of LDHA through deubiquitination and maintains LDHA protein stability	LDHA protein stability is maintained, MAPK/AKT-related signaling and cell proliferation are promoted, whereas the direct effect on glycolytic flux remains insufficiently validated.	PTC; PTC cells and patient tumor tissues	[30]
USP5	PFKP	Inhibits PFKP protein degradation through deubiquitination and increases PFKP stability	Promotes glucose uptake and lactate/pyruvate production and enhances aerobic glycolysis	TNBC; MDA-MB-231 and SUM159 cells and nude mouse xenografts	[24]
USP7	PKM2	Removes K48-linked polyubiquitin chains from PKM2 and increases PKM2 protein stability	Regulates PKM2 protein stability; however, the direct effect on glycolysis in specific cancer types has not been fully validated	HEK293T and HeLa cells; mainly molecular deubiquitination experiments	[26]
USP7	c-Myc	Reduces c-Myc ubiquitination and inhibits its protein degradation	Increases c-Myc stability, promotes GLUT1, HK2, and LDHA expression, and enhances glucose uptake, lactate production, and aerobic glycolysis	PDAC; AsPC-1, CFPAC-1, and PANC-1 cells and animal models	[13]
USP7	c-Abl	Removes K48-linked polyubiquitin chains from c-Abl and inhibits its proteasomal degradation	Promotes cytoplasmic accumulation of c-Abl, enhances HK2 phosphorylation, protein stability, and catalytic activity, thereby increasing lactate and pyruvate production	NSCLC; A549, H1299, and H460 cells and nude mouse xenografts	[48]
USP7	FKBP4	Removes polyubiquitin chains from FKBP4 and increases its protein stability	Regulates PI3K/AKT-related signaling, promotes GLUT1 and LDHA expression, enhances glycolysis, and participates in osimertinib resistance	Osimertinib-resistant NSCLC; PC9/OR and H1975/OR cells and nude mouse xenografts	[41]
USP10	PGK1	Promotes PGK1 deubiquitination and increases its stability under KIF15-mediated protein recruitment	Maintains PGK1 protein stability, promotes glucose uptake and lactate production, and enhances glycolysis and tumor cell proliferation	PC; MIA PaCa-2 and PANC-1 cells and animal models	[32]
USP13	PTEN	Removes polyubiquitin chains from PTEN and inhibits its protein degradation	Stabilizes PTEN to inhibit PI3K/AKT signaling, reduces glycolytic activity, and suppresses tumor growth	BC; MCF7, SUM159, BT549, and MDA-MB-231 cells and xenograft models	[39]
USP13	PTEN	Regulates PTEN protein levels and PTEN/AKT signaling	Inhibits AKT signaling and GLUT1/HK2 expression, reduces glucose uptake and lactate production, and decreases glycolytic levels	OSCC; CAL27 and HSC4 cells and nude mouse xenografts	[40]
USP14	GLUT1	Removes K48-linked polyubiquitin chains from GLUT1, with K245 as an important ubiquitination site	Inhibits GLUT1 protein degradation and enhances glucose uptake and tumor metabolic competition	HCC; HCC cells, patient tissues, and immunocompetent mouse liver cancer models	[37]
USP17	c-Myc	Removes K48-linked polyubiquitin chains from c-Myc and inhibits proteasomal degradation	Increases c-Myc stability, promotes HK2 and LDHA expression, increases glucose consumption and lactate production, and enhances glycolysis	Prostate cancer; mainly PC-3 cells for glycolysis experiments	[42]
USP19	HIF-1α	Maintains HIF-1α stability through a non-catalytic protein-protection mechanism	Promotes HIF-1α accumulation and expression of target genes such as GLUT1 under hypoxia, possibly leading to hypoxic metabolic adaptation; however, changes in glycolytic flux have not been directly validated	Basal hypoxia-response models; HeLa, U2OS, and HEK293T cells	[51]
USP20	PKM2	Catalytically removes ubiquitin modifications from PKM2	Modulates the ubiquitination level of PKM2, but no obvious increase in PKM2 protein abundance was observed, and changes in glycolytic activity were not confirmed	HeLa cells; mainly molecular deubiquitination experiments	[25]
USP20	RAB8A (GDP-bound)	Selectively removes K48-linked polyubiquitin chains from inactive RAB8A and promotes its protein accumulation	Restricts GLUT1 translocation to the plasma membrane by interfering with the RAB8A activation cycle, reducing glucose uptake and lactate production and inhibiting glycolysis-related metabolism	PDAC; PDAC cells, animal models, and KPC mouse-derived primary tumor cells	[38]
USP28	FOXC1 (protein stability target)	Inhibits ubiquitin–proteasome-dependent degradation of FOXC1	Increases FOXC1 protein stability, promotes aerobic glycolysis and CRC cell proliferation	CRC; CRC cell models	[53]
USP29	HIF-1α	Inhibits HIF-1α protein degradation through direct deubiquitination	Increases HIF-1α stability and transcriptional activity, promotes HK2 expression, enhances aerobic glycolysis, and mediates sorafenib resistance	HCC; sorafenib-resistant HLE, Hep3B, and Huh7 cells and nude mouse xenografts	[45]
USP35	PKM2	Maintains PKM2 protein stability by inhibiting ubiquitin-dependent degradation of PKM2 through deubiquitination	Promotes glucose uptake, lactate and pyruvate production, and enhances glycolysis	HCC; Huh7 and Hep3B cells and clinical HCC tissues	[28]
USP43	c-Myc	Removes K48-linked polyubiquitin chains at K148 and K289 of c-Myc; also impedes FBXW7-mediated degradation through competitive binding	Increases c-Myc stability and transcriptional activity, promotes expression of GLUT1, HK2, PKM2, and LDHA, and enhances aerobic glycolysis	BLCA; T24, 5637, and UM-UC-3 cells and nude mouse xenograft models	[44]
USP43	14-3-3 proteins and HIF-1α-related complexes	Promotes nuclear accumulation of HIF-1α-related complexes through non-catalytic protein–protein interactions, independent of increased HIF-1α stability	Enhances HIF-1α nuclear localization and transcription of hypoxia-related target genes, promoting glycolytic adaptation to hypoxia	Multiple cancer cell models, mainly HeLa, also A549 and RCC4 cells	[52]
USP46	PTBP1	Removes K48-linked polyubiquitin chains from PTBP1 and inhibits proteasomal degradation	Maintains PTBP1 stability, promotes alternative splicing of PKM, increases the PKM2/PKM1 expression ratio, enhances glycolysis, and promotes tamoxifen resistance	ER^+^ breast cancer (BC); MCF-7 and T47D cells and their corresponding tamoxifen-resistant derivatives	[47]
USP54	p53	Reduces p53 ubiquitination levels and inhibits its protein degradation	Increases p53 stability, inhibits GLUT1 expression and glucose uptake, reduces aerobic glycolysis, and promotes mitochondrial oxidative metabolism	LUAD; A549 and A427 cells and animal models	[46]
UCHL3	PKM2	Removes K48-linked polyubiquitin chains from PKM2 and inhibits its proteasomal degradation	Increases PKM2 stability, promotes glucose uptake and lactate production, enhances glycolysis and tumor cell proliferation	BLCA; T24 and 5637 cells and nude mouse xenografts	[29]
JOSD2	ALDOA, PFK1, PHGDH	Maintains the stability and activity of multiple metabolic enzymes through deubiquitination	Enhances ALDOA- and PFK1-related glycolytic activity and, through PHGDH, coordinates the shunting of glycolytic intermediates toward the serine biosynthesis pathway	Multiple tumor models, with NSCLC studies constituting an important component; A549 and other cell lines, as well as animal models	[31]
OTUB1	HIF-1α	Non-catalytically inhibits E2-mediated ubiquitin transfer, reducing K48-linked polyubiquitination of HIF-1α	Increases HIF-1α stability and transcriptional activity, promotes expression of metabolism-related genes such as PKM2, and enhances glucose uptake and compensatory glycolysis under hypoxia	NSCLC; mainly based on the H1299 cell hypoxia model	[50]
OTUB2	PKM2	Reduces PKM2 ubiquitination by blocking the binding of the E3 ligase Parkin to PKM2	Increases PKM2 enzymatic activity, promotes glucose consumption, lactate production, and ATP generation, and enhances glycolysis	CRC; colorectal cancer cell models, including HCT116 cells, and mouse xenograft models.	[49]
PSMD14	PKM2	Removes K63-linked polyubiquitin chains from PKM2, regulating its oligomeric state without significantly altering protein abundance	Reduces PKM2 tetramer formation, decreases its pyruvate kinase activity, and promotes nuclear translocation, thereby promoting aerobic glycolytic reprogramming in ovarian cancer	OC; OVCAR-3 and other ovarian cancer cells, as well as nude mouse xenografts	[33]
PSMD14	LDHA	Removes K48- and K63-linked ubiquitin chains from LDHA and increases LDHA protein stability	Promotes lactate accumulation and histone H3K18 lactylation, increases ACLY transcription and fatty acid synthesis, and drives glycolytic and lipid metabolic reprogramming	PC; PANC-1 and MIA PaCa-2 cells and patient-derived xenografts	[34]

**Table 2:** Tumor Model-Based Evidence for DUB-Mediated Regulation of Glycolysis Through Metabolic Signaling Pathways.

Signaling Pathway	DUB/Molecular Axis	Major Mechanism	Glycolytic Effect	Significance & Reference
mTOR pathway	USP10 deficiency–mTOR/S6K–HIF-1α	USP10 deficiency enhances mTOR/S6K signaling and increases HIF-1α protein synthesis	Enhances glycolytic capacity and glycolytic reserve	Colorectal cancer cell metabolism and malignant phenotypes [56]
mTOR pathway	OTUB2-U2AF2-AKT/mTOR	OTUB2 stabilizes U2AF2, activates AKT/mTOR	Enhances Warburg effect	NSCLC proliferation and metastasis [57]
mTOR pathway	USP14-PBX3-AKT/mTOR	USP14 stabilizes PBX3, activates AKT/mTOR	Promotes glycolysis	Breast cancer proliferation and metastasis [58]
AMPK pathway	USP10-AMPKα	USP10 deubiquitinates AMPKα to promote its activation; USP10 loss impairs AMPK activation	Upregulates ALDOA, LDHA, PDK1, enhances glycolysis	Metabolic disorders and tumor reprogramming [62]
HIF-1 pathway	USP25-HIF-1α	USP25 stabilizes HIF-1α	Maintains glycolytic flux	Pancreatic ductal adenocarcinoma growth [19]
HIF-1 pathway	USP43-HIF-1α	Promotes HIF-1α nuclear accumulation and enhances transcriptional activity	Enhances HIF-1 transcriptional response under hypoxia	Tumor hypoxic adaptation [52]
HIF-1 pathway	USP21-HSP90-HIF-1α	USP21 stabilizes HSP90, indirectly upregulates HIF-1α	Upregulates glycolytic genes	Cholangiocarcinoma cell proliferation [68]
HIF-1 pathway	USP28-PKM2-HIF-1α	USP28 deubiquitinates PKM2, thereby enhancing PKM2-dependent HIF-1α signaling	Promotes glycolysis	Cholangiocarcinoma progression [69]
HIF-1 pathway	USP29-HIF-1α	Removes ubiquitin chains from HIF-1α, prevents degradation	Sustains hypoxic glycolysis	Sorafenib resistance in HCC [45]
HIF-1 pathway	USP11-HIF-1α-PDK1/LDHA	Stabilizes HIF-1α and activates PDK1/LDHA	Enhances glycolysis	HCC proliferation and metastasis [70]
HIF-1 pathway	OTUB1-HIF-1α	Stabilizes HIF-1α via non-canonical ubiquitination-inhibitory activity, independent of the canonical PHD/VHL pathway	Promotes hypoxic glycolytic reprogramming	Tumor hypoxic adaptation [50]
HIF-1 pathway	USP22-HIF-1α	Stabilizes HIF-1α under hypoxia	Maintains glycolysis, stemness, and drug resistance	Chemoresistance in nasopharyngeal carcinoma [71]
Wnt/β-catenin pathway	UCHL5-β-catenin	Inhibits ubiquitin-mediated degradation of β-catenin	Enhances glycolysis	HCC proliferation and metastasis [72]
Notch1 pathway	USP24-PLK1-Notch1	USP24 stabilizes PLK1, activates Notch1	Promotes glycolysis	Poor prognosis in gastric cancer [73]
PTEN/AKT pathway	USP13-PTEN-AKT	Upregulates PTEN, inhibits AKT signaling	Suppresses glycolysis	Tumor-suppressive effects [39]
Wnt/c-Myc pathway	USP7-β-catenin-c-Myc	USP7 stabilizes β-catenin, upregulates c-Myc	Promotes glycolysis	Pancreatic cancer metabolic reprogramming [13]
PI3K/AKT pathway	OTUB2-PKM2-PI3K/AKT	Activates PKM2/PI3K/AKT signaling	Promotes glycolysis	Endometrial cancer proliferation and invasion [75]
STAT3/FOXO1 pathway	USP21-STAT3/FOXO1	Activates STAT3/FOXO1 signaling	Promotes glycolysis	Tumor cell proliferation [76]

## Data Availability

No new data were created or analyzed in this study. Data sharing is not applicable to this article.

## Abbreviations

2-DG	2-deoxy-D-glucose
ACSS2	acyl-CoA synthetase short chain family member 2
ALDOA	aldolase A
ALDOC	aldolase C
AMPK	AMP-activated protein kinase
ARG-1	arginase-1
ATRAP	AT1 receptor-associated protein
CARM1	coactivator-associated arginine methyltransferase 1
CCA	cholangiocarcinoma
CRC	colorectal cancer
CSC	cancer stem cell
CSF	colony-stimulating factor
DUBs	deubiquitinases
ENO2	enolase 2
ENO3	enolase 3
FOXC1	forkhead box C1
G6PC	glucose-6-phosphatase catalytic subunit
GLUT1	glucose transporter 1
GLUTs	glucose transporters
GPR81	G protein-coupled receptor 81
HCC	hepatocellular carcinoma
HIF-1	hypoxia-inducible factor 1
HIF-1α	hypoxia-inducible factor 1α
HK2	hexokinase 2
HSP90	heat shock protein 90
IL-15	interleukin-15
JOSD2	Josephin domain-containing protein 2
LDHA	lactate dehydrogenase A
MCT1	monocarboxylate transporter 1
MDSCs	myeloid-derived suppressor cells
NF-κB	nuclear factor kappa-B
NK	natural killer
NSCLC	non-small cell lung cancer
O-GlcNAcylation	O-linked N-acetylglucosamine modification
OGT	O-GlcNAc transferase
OTUB1	OTU deubiquitinase, ubiquitin aldehyde binding 1
OTUB2	OTU deubiquitinase OTUB2
PBX3	pre-B-cell leukemia transcription factor 3
PDAC	pancreatic ductal adenocarcinoma
PDH	pyruvate dehydrogenase
PDK1	pyruvate dehydrogenase kinase 1
PD-L1	programmed death-ligand 1
PFK-1	phosphofructokinase-1
PFKP	phosphofructokinase platelet
PGK1	phosphoglycerate kinase 1
PKM2	pyruvate kinase M2
PLK1	Polo-like kinase 1
PTBP1	polypyrimidine tract-binding protein 1
PTEN	phosphatase and tensin homolog
SIRT7	sirtuin 7
TAMs	tumor-associated macrophages
TCA	tricarboxylic acid
TME	tumor microenvironment
TNBC	triple-negative breast cancer
TWIST1	Twist-related protein 1
U2AF2	U2 small nuclear RNA auxiliary factor 2
UCHL5	ubiquitin carboxyl-terminal hydrolase L5
USF1	upstream stimulatory factor 1
USP3	ubiquitin-specific protease 3
USP5	ubiquitin-specific protease 5
USP7	ubiquitin-specific protease 7
USP10	ubiquitin-specific protease 10
USP11	ubiquitin-specific protease 11
USP13	ubiquitin-specific protease 13
USP14	ubiquitin-specific protease 14
USP17	ubiquitin-specific protease 17
USP22	ubiquitin-specific protease 22
USP24	ubiquitin-specific protease 24
USP25	ubiquitin-specific protease 25
USP28	ubiquitin-specific protease 28
USP29	ubiquitin-specific protease 29
USP38	ubiquitin-specific protease 38
USP39	ubiquitin-specific protease 39
USP43	ubiquitin-specific protease 43
USP46	ubiquitin-specific protease 46
USP54	ubiquitin-specific protease 54
USP9X	ubiquitin-specific protease 9X
VEGF	vascular endothelial growth factor
WDR1	WD repeat domain 1
c-Myc	cellular myelocytomatosis oncogene
mTORC1	mammalian target of rapamycin complex 1
